# Genome-wide dynamic transcriptional profiling in *clostridium beijerinckii *NCIMB 8052 using single-nucleotide resolution RNA-Seq

**DOI:** 10.1186/1471-2164-13-102

**Published:** 2012-03-20

**Authors:** Yi Wang, Xiangzhen Li, Yuejian Mao, Hans P Blaschek

**Affiliations:** 1 Department of Agricultural and Biological Engineering, University of Illinois at Urbana-Champaign, Urbana, IL 61801, USA; 2 Institute for Genomic Biology, University of Illinois at Urbana-Champaign, Urbana, IL 61801, USA; 3 Department of Animal Sciences, University of Illinois at Urbana-Champaign, Urbana, IL 61801, USA; 4 Chengdu Institute of Biology, Chinese Academy of Sciences, Chengdu Sichuan 610041, China; 5 Department of Food Science and Human Nutrition, University of Illinois at Urbana-Champaign, Urbana, IL 61801, USA; 6 Center for Advanced Bioenergy Research (CABER), University of Illinois at Urbana-Champaign, Urbana, IL 61801, USA

## Abstract

**Background:**

*Clostridium beijerinckii *is a prominent solvent-producing microbe that has great potential for biofuel and chemical industries. Although transcriptional analysis is essential to understand gene functions and regulation and thus elucidate proper strategies for further strain improvement, limited information is available on the genome-wide transcriptional analysis for *C. beijerinckii*.

**Results:**

The genome-wide transcriptional dynamics of *C. beijerinckii *NCIMB 8052 over a batch fermentation process was investigated using high-throughput RNA-Seq technology. The gene expression profiles indicated that the glycolysis genes were highly expressed throughout the fermentation, with comparatively more active expression during acidogenesis phase. The expression of acid formation genes was down-regulated at the onset of solvent formation, in accordance with the metabolic pathway shift from acidogenesis to solventogenesis. The acetone formation gene (*adc*), as a part of the *sol *operon, exhibited highly-coordinated expression with the other *sol *genes. Out of the > 20 genes encoding alcohol dehydrogenase in *C. beijerinckii*, Cbei_1722 and Cbei_2181 were highly up-regulated at the onset of solventogenesis, corresponding to their key roles in primary alcohol production. Most sporulation genes in *C. beijerinckii *8052 demonstrated similar temporal expression patterns to those observed in *B. subtilis *and *C. acetobutylicum*, while sporulation sigma factor genes *sigE *and *sigG *exhibited accelerated and stronger expression in *C. beijerinckii *8052, which is consistent with the more rapid forespore and endspore development in this strain. Global expression patterns for specific gene functional classes were examined using self-organizing map analysis. The genes associated with specific functional classes demonstrated global expression profiles corresponding to the cell physiological variation and metabolic pathway switch.

**Conclusions:**

The results from this work provided insights for further *C. beijerinckii *strain improvement employing system biology-based strategies and metabolic engineering approaches.

## Background

Solvents such as acetone, butanol and ethanol (ABE) produced through microbial fermentation represent important potential renewable fuels and chemicals [1]. *Clostridium acetobutylicum *and *C. beijerinckii *are among the prominent solvent-producing species. *C. beijerinckii *exhibits a broader substrate range and optimum pH for growth and solvent production [2]; thus it may have greater potential for biosolvent production than *C. acetobutylicum*.

The genome of *C. beijerinckii *NCIMB 8052 was sequenced by the DOE Joint Genome Institute in 2007. The genome size is 6.0 Mb, which is 50% larger than that of *C. acetobutylicum *ATCC 824. The *C. beijerinckii *8052 solvent-producing genes are all located on the chromosome, as opposed to the location of these genes on a mega-plasmid in *C. acetobutylicum *824. Transcriptional analysis is essential to understand gene functions and regulation and thus elucidate proper strategies for further strain improvement. While global gene expression patterns for *C. acetobutylicum *have been studied extensively [3-9], limited information is available on the genome-wide transcriptional analysis for *C. beijerinckii *[10]. RNA-Seq is based on the high-throughput DNA sequencing technology and provides an approach to profile and quantify gene expression to an unprecedented resolution and depth [11-14]. RNA-Seq technology has several advantages over DNA microarray methods for transcriptional analysis. First, microarray and other hybridization techniques exhibit a relatively low dynamic range for the detection of transcriptional levels due to background, saturation and poor sensitivity for gene expression at very low or high levels [15], while RNA-Seq does not have an upper limit for quantification and has a larger dynamic range of expression levels. In addition, when compared to microarray-based approaches, RNA-Seq has very low background noise because DNA sequence reads can be unambiguously mapped to unique regions along the genome [16]. Several recent studies have proven that RNA-Seq is a powerful tool for transcriptional analysis [12,17,18]. In this study, a genome-wide transcriptional analysis of *C. beijerinckii *8052 over the course of a batch fermentation was described from an unbiased perspective using RNA-Seq technology. The findings from this work provided insights for further *C. beijerinckii *strain improvement employing system biology-based strategies and metabolic engineering. Furthermore, this work is also an essential methodology reference for conducting transcriptional analysis employing next-generation sequencing technology.

## Results and discussion

### Growth kinetics and ABE fermentation

**Figure 1:**
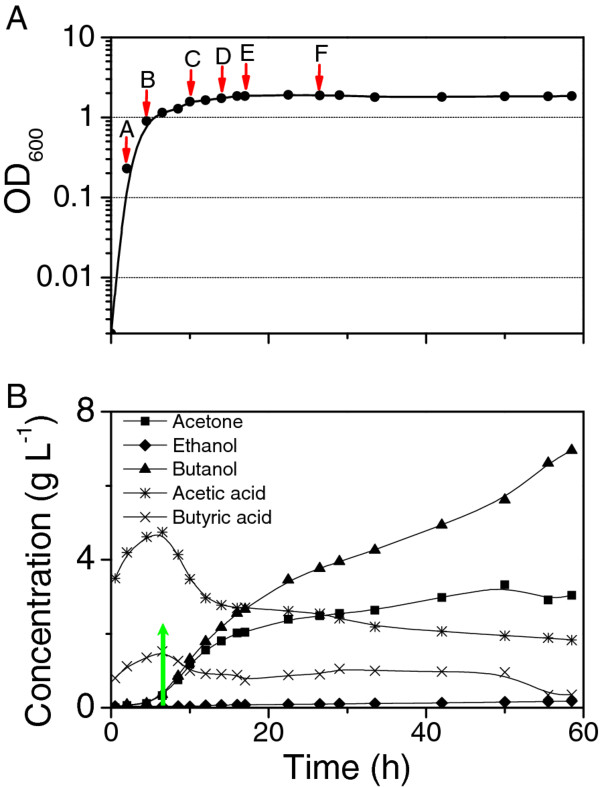
**Fermentation kinetics of *C. beijerinckii *8052 batch culture**. (A) Cell growth curve with sampling points for RNA-Seq indicated by red arrows. (B) Solvents and acids production over time with the onset of solvent production indicated by a green arrow.

The *C. beijerinckii *8052 growth response in the batch fermentation is illustrated in Figure 1A (see also [19]). The fermentation shifted from acidogenesis to solventogenesis at approximately 4.5-8 h. Production of solvents was detected at between 4.5-6.5 h after the start of fermentation, which corresponded to the late exponential growth phase. Butanol continued to increase throughout the stationary phase (Figure 1B). Samples for RNA isolation were collected at various time points during acidogenesis (2 and 4.5 h) and solventogenesis (after 6.5 h) (Figure 1A). These samples were sequenced for dynamic transcriptional profiling for *C. beijerinckii *8052.

### Overall gene transcription dynamics

The 75-nt sequence reads were mapped to the *C. beijerinckii *8052 genome. Only those reads that mapped unambiguously to the genome were used for further analyses (Table 1). Based on RNA-Seq sequence data, 3386 out of 5020 (67.4%) protein-coding genes had detectable expression in all six sampling time points, while 78 demonstrated no transcripts over all six time points, and are likely silent [19].

**Table 1:** Summary of RNA-Seq sequencing and data analysis results

Sample	A	B	C	D	E	F*	Total
Time collected (h)	2	4.5	10	14	17	26.5	

Total number of reads	8988633	9457480	8011531	8448929	10363535	38574501	83844609

No. of reads mapped	8473125	9037616	7514804	7730815	9842491	37676913	80275764

No. of reads unambiguously mapped	6776544	7274568	6096405	6189652	8096169	35027722	69461060

No. of genes with detectable expression**	4219	4082	4496	4453	4487	4750	5024

Range in expression levels (RPKM)	3.2 × 10^-1 ^~ 2.5 × 10^4^	5.8 × 10^-2 ^~ 6.0 × 10^4^	4.5 × 10^-2 ^~ 2.5 × 10^4^	7.0 × 10^-2 ^~ 9.0 × 10^4^	1.0 × 10^-1 ^~ 8.6 × 10^4^	3.6 × 10^-2 ^~ 9.8 × 10^4^	3.6 × 10^-2 ^~ 9.8 × 10^4^

*Sample F was sequenced with 1 sample/lane, while samples A-E with 2 samples/lane. See Methods section for more details; ** Pseudogenes included

On the other hand, there were some genes that demonstrated little variation in expression levels throughout the entire fermentation process, and they are regarded as putative housekeeping genes (HKGs). In this study, 177 protein-coding genes were identified as putative HKGs with the lowest coefficient of variation (CV) in RPKM (reads/Kb/Million, see the Methods section) values among all the sampling points [19]. For accurate gene expression analysis, normalization of gene expression against HKGs is generally required. In the qRT-PCR test in this work, a putative HKG (Cbei_2428, encoding peptidase T) with highly constant expression levels across all six sampling points was selected as the endogenous control gene.

**Figure 2:**
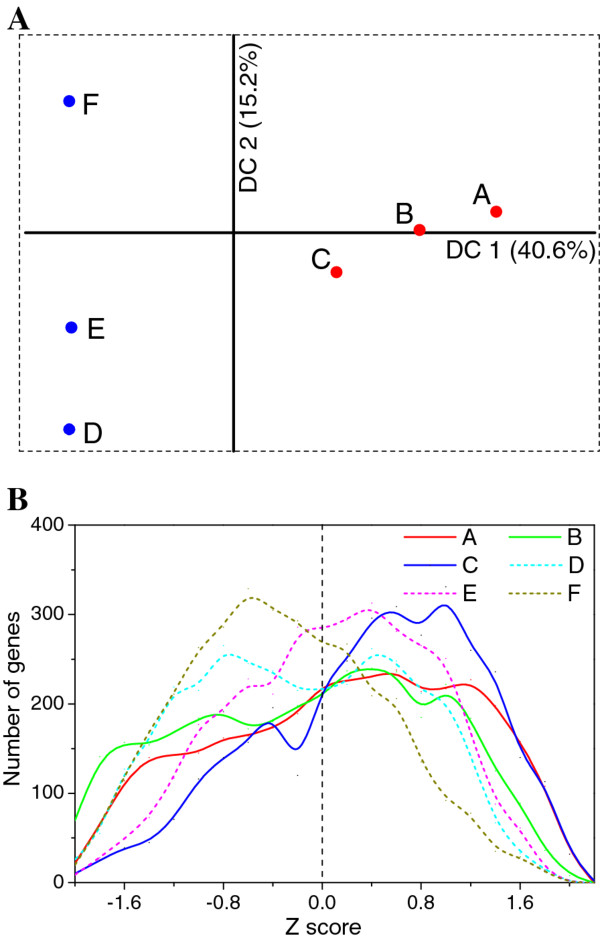
**Overall gene transcription profiles during the fermentation based on RNA-Seq data**. (A) Detrended Correspondence Analysis (DCA). Sampling points from exponential phase and transition phase (A-C) were indicated in red, while points from stationary phase (D-F) were indicated in blue. (B) Gene distribution based on Z-score analysis. Samples A-F were indicated by different line styles and colors.

Detrended Correspondence Analysis (DCA) was conducted on the RNA-Seq data to generate overall dynamic transcription profiles throughout the batch fermentation process. DCA is an ordination technique that uses detrending to remove the 'arch effect' usually existing in correspondence analysis [20]. As shown in Figure 2A, an obvious temporal variation trend was observed for the overall gene transcription. While the first three samples which were from the exponential and transition phases presented both significant first detrended correspondence (DC1, explained 40.6% of detrended correspondence) and second detrended correspondence (DC2, explained 15.2% of detrended correspondence), the last three samples which were from stationary phase demonstrated only significant DC2 but little DC1. The temporal variation trend of the overall gene expression is consistent with different cell physiological states during transition from acidogenesis to solventogenesis.

A Z-score analysis was conducted to investigate the overall gene expression pattern at each time point. Z-score measures the number of standard deviations that a gene expression in a specific sample is from the mean expression of this gene over all samples. It is a dimensionless quantity derived by subtracting the mean expression of a specific gene over all samples from the individual expression level in a specific sample and then dividing the difference by the standard deviation [18,21]. The results demonstrated temporally dynamic patterns over the course of cell growth and batch fermentation (Figure 2B). Generally, during exponential and early stationary phases (2-10 h), more genes shifted towards higher expression values (to the right on Figure 2B), while during late stationary phases, more genes shifted to lower expression levels (to the left on Figure 2B). A list of genes whose Z-score was > 1.7 in each sample is summarized in Additional file 1: Table S1. The highly expressed gene categories were different at different time points during the course of the fermentation. For example, translation related genes (COG J) were predominant (49 out of total 141 genes in this list) in sample A (at 2 h), indicating fast protein synthesis during this growth stage; while signal transduction mechanism involved genes (COG T) were overrepresented at 10 h, indicating the cellular reaction to the environmental changes.

**Figure 3:**
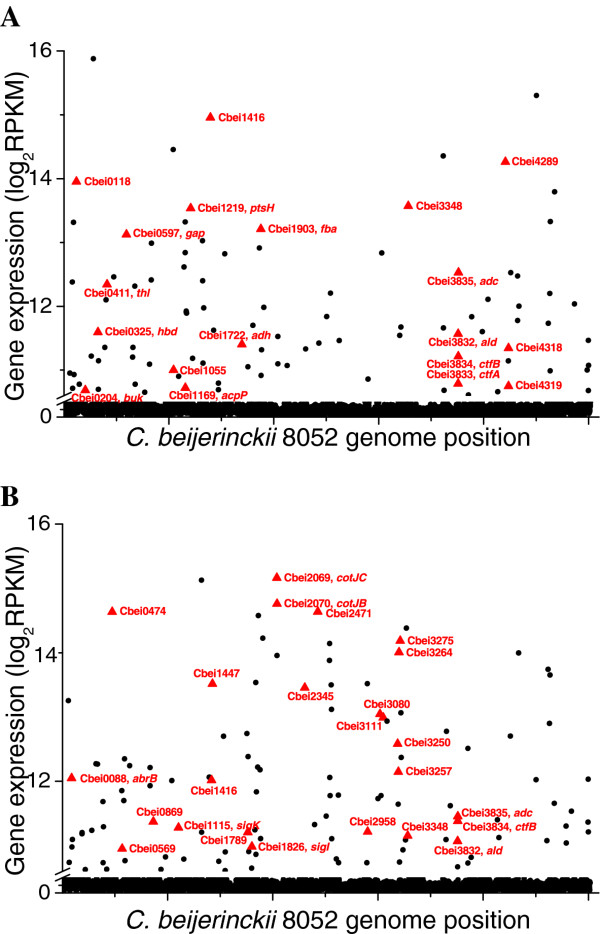
**Identification of highly expressed genes in samples B and D by plotting the log_2_-scaled gene expression values against the gene loci through the genome**. (A) Plot of gene expression level (log_2_(RPKM)) against the gene locus through the genome in sample B (at 4.5 h). (B) Plot of gene expression level (log_2_(RPKM)) against the gene locus through the genome in sample D (at 14 h). The highly expressed genes involved in the main metabolic pathways during the fermentation were labeled with larger triangles.

The expression of different genes in a specific sample can be compared by plotting log_2_-transformed RPKM values of all genes against their loci along the genome (Figure 3).

Sample B represented the beginning of the transition from acidogenesis to solventogenesis. In this sample, among the highest expressed genes, besides the genes encoding hypothetical proteins (31 out of the top 100 highest expressed), 12 are ribosomal protein genes, indicating active protein synthesis machinery in the cell at this time point. In addition, genes involved in acid and solvent formation were detected as highly expressed, including *adc *(Cbei_3835), *thl *(Cbei_0411), *hbd *(Cbei_0325), *ald *(Cbei_3832), *adh *(Cbei_1722), *ctfB *(Cbei_3834), *ctfA *(Cbei_3833) and *buk *(Cbei_0204). Highly expressed genes also included those encoding proteins that mediate electron transfer, such as Cbei_1416 (rubrerythrin), Cbei_0118, Cbei_3348 (desulfoferrodoxin), Cbei_4318, Cbei_4319 (flavodoxin), and those genes related to cell motility (Cbei_4289), phosphotransferase system (PTS) for sugar uptake (Cbei_1219) and glycolytic activity (Cbei_1903, *fba *and Cbei_0597, *gap*) (Figure 3 and Additional file 2: Table S2). The time frame of sample D was consistent with the transition to non-active growth and clostridial spore formation. While the solventogenesis genes (*adc, ald, ctfB, ctfA*) and several ferredoxin activity protein encoding genes (Cbei_3257, _3348 and _0569) were still actively expressed at this stage (Figure 3 and Additional file 2: Table S2), genes encoding sporulation-related proteins demonstrated the highest transcriptional activities. These genes included those encoding spore coat proteins (Cbei_2069, *cotJC *and Cbei_2070, *cotJB*), small acid-soluble spore proteins (Cbei_2471, _0474, _3275, _3264, _1447, _2345, _3080, _3111 and _3250), AbrB family transcriptional regulator (Cbei_0088, annotated as AbrB family stage V sporulation protein T in *C. acetobutylicum*) and sporulation sigma factor SigK (Cbei_1115).

It is interesting that the hypothetical genes account for a large fraction of the highly expressed genes (in both samples B and D presented above). As we mentioned previously [19], the genome annotation for *C. beijerinckii *8052 is far from completed. Many of the hypothetical genes predicted by computational analysis algorithms may have important functions for the cell activities. The RNA-Seq results herein provided useful references for future studies in defining the specific functions of these genes.

### Expression of primary metabolic genes

#### Glycolysis genes

**Figure 4:**
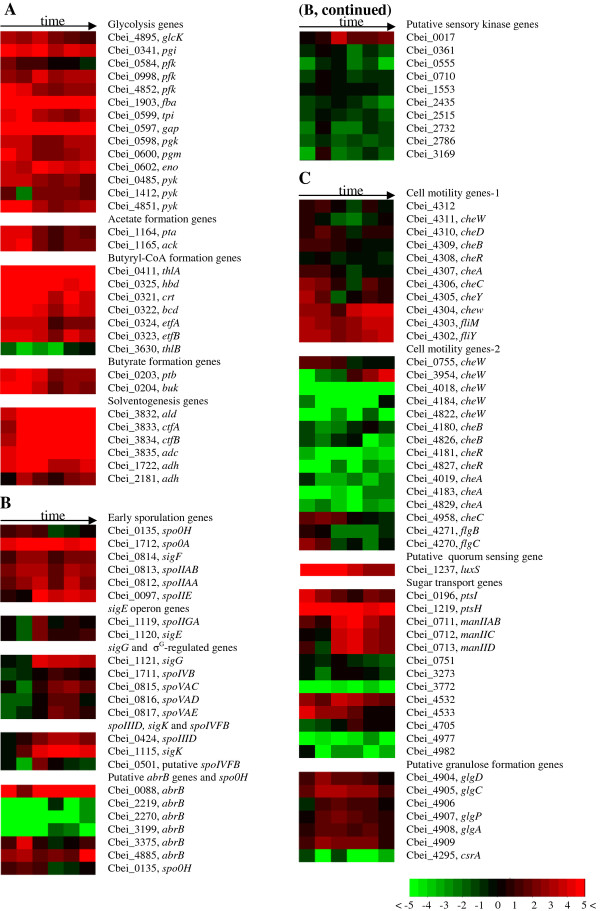
**Time course expression profiles of important genes during the batch fermentation process**. (A) Genes related to glycolysis, acidogenesis and solventogenesis. (B) Sporulation genes, putative *abrB *genes and putative sensory kinase genes. (C) Genes related to cell motility, putative quorum sensing, sugar transport and granulose formation.

Generally, the glycolysis genes encoding the pathway for conversion of glucose to pyruvate were expressed at high levels throughout the entire fermentation process (Figure 4A). Comparatively, the expression of most of these genes decreased slightly after entering the stationary phase, down-regulated by 2- to 4-fold. In *C. beijerinckii *8052, three genes (Cbei_0584, Cbei_0998 and Cbei_4852) are annotated as encoding 6-phosphofructokinase (pfk). While Cbei_0584 and _4852 were down-regulated 2.3- and 2.7-fold respectively after entering stationary phase, Cbei_0998 was up-regulated 3.8-fold at time point C and expressed at higher levels throughout stationary phase. Cbei_0485, Cbei_1412 and Cbei_4851 are all annotated as encoding pyruvate kinase (pyk). While Cbei_0485 and _4851 decreased by 2.5-fold after entering stationary phase, Cbei_1412 was expressed at a higher level throughout stationary phase when compared to the exponential phase. These observations imply that different allelic genes may be induced at different specific stages and play different roles during the fermentation process. The expression patterns of glycolysis genes in this study are similar to those found for *C. beijerinckii *8052 in Shi and Blaschek (2008) [10]. While in a time course transcriptional analysis of *C. acetobutylicum *824 during a batch fermentation, most of the glycolysis genes showed higher expression during stationary-phase [5].

#### Acidogenesis and solventogenesis genes

The acetate formation genes encoding phosphotransacetylase (*pta*, Cbei_1164) and acetate kinase (*ack*, Cbei_1165) and the butyrate formation genes encoding phosphate butyryltransferase (*ptb*, Cbei_0203) and butyrate kinase (*buk*, Cbei_0204) were up-regulated during the acidogenesis phase and peaked at time point B, and declined 2- to 4-fold at the onset of solvent formation (Figure 4A). Based on the sequencing data, the *pta*-*ack *and *ptb-buk *gene operon structures were identified [19]. The coordinated expression patterns of these two gene clusters further confirmed this transcriptional organization. Expression of genes encoding proteins involved in butyryl coenzyme A (butyryl-CoA) formation was induced early during the acidogenesis phase, and remained at high levels after cells entered stationary phase (time point C), and afterwards decreased 3-to 7-fold at late stationary phase, although the absolute transcription level was still high (Figure 4A).

The product of the *thlA *(Cbei_0411) in *C. beijerinckii *has 77% amino acid sequence identity to the product of *thlA *(CAC2873) in *C. acetobutylicum*, and the product of a second thiolase gene (*thlB*, Cbei_3630) shows 90% amino acid sequence identity to that of *thlB *(or *thil*) in *C. acetobutylicum *(CAP0078). A transcriptional analysis with qRT-PCR for continuous cultures of *C. acetobutylicum *indicated that the level of *thlB *transcripts was much lower compared to the level of *thlA *transcript, and the expression of *thlB *was maximal at about 10 to 15 h after induction of solvent formation [22]. Whereas another transcriptional analysis for continuous cultures of *C. acetobutylicum *showed that *thlB *was highly induced only during the transition state from acidogenesis to solventogenesis [23]. In this study, the expression level of *thlB*, although much lower than that of *thlA*, was found to be maximal in late stationary phase (time points E and F), which is around 10 to 20 h after onset of solventogenesis (Figure 1). While thlA is involved in the primary metabolism of acid and solvent formation, the physiological function of thlB warrants further study. In the *C. beijerinckii *8052 genome, there are different genes encoding for isoenzymes active in specific stages and playing different roles. Transcriptomic analysis leads to insights for further biochemical characterization of the isoenzymes properties and functions.

In *C. acetobutylicum *824, the genes *adhE *(bifunctional acetaldehyde-CoA/alcohol dehydrogenase, CA_P0162), *ctfA *(acetoacetyl-CoA: acetate/butyrate-CoA transferase subunit A, CA_P0163) and *ctfB *(acetoacetyl-CoA: acetate/butyrate-CoA transferase subunit B, CA_P0164) are located in the *sol *(solvent formation) operon on the mega-plasmid pSOL1, while *adc *(acetoacetate decarboxylase, CA_P0165) is organized in a monocistronic operon in the opposite direction [24-26]. The regulation of *adc *was believed to differ from that of the *sol *operon, and the expression of *adc *was reported to be initiated earlier than the *sol *operon in both batch and continuous cultures of *C. acetobutylicum *[5,23,27-29]. Based on our sequencing data, a *sol *operon organized in the order of *ald *(Cbei_3832, encoding aldehyde dehydrogenase)-*ctfA *(Cbei_3833)-*ctfB *(Cbei_3834)-*adc *(Cbei_3835) was revealed in *C. beijerinckii *8052 [19]. Consistent with this transcriptomic organization, coordinated expression was observed for the *sol *operon genes. Their expression was up-regulated at the onset of solventogenesis phase and maintained at high levels during the stationary phase (Figure 4A).

Two iron-containing alcohol dehydrogenase genes *adhA *(GenBank: AF497741.2) and *adhB *(GenBank: AF497742.1) were previously characterized in the solvent-producing strain *C. beijerinckii *NRRL B592 [10,30]. The enzymes encoded by both genes were classified as primary alcohol dehydrogenases, which catalyze the reaction for primary alcohol (that is, butanol and ethanol, but not isopropanol) production [30]. The product of Cbei_2181 in *C. beijerinckii *8052 exists 99% and 97% amino acid sequence identity to that of *adhA *and *adhB*, and the product of Cbei_1722 has 97% and 94% amino acid sequence identity to that of *adhB *and *adhA*, respectively. Cbei_2181 and _1722 may play key roles during primary alcohol production in *C. beijerinckii *8052. Both genes were induced to high levels (9 and 4-fold up respectively) when the fermentation transitioned from the acid production phase to the solvent production phase (time point B), and then decreased. Similar transient up-regulation of *adh *genes and enzymes associated with active solvent production was previously reported for *C. beijerinckii *NRRL B592 and *C. acetobutylicum *824 [30-32].

There are 20 annotated alcohol dehydrogenase genes in *C. beijerinckii *8052, suggesting the great potential for solvent production of this microorganism. Most of these genes were induced early during the acidogenesis phase and expressed at high levels throughout the solvent production process, although some of them did not have detectable expression at some specific time points (Additional file 3: Table S3). The specific functions of different alcohol dehydrogenase genes associated with *C. beijerinckii *require further investigation.

#### Sporulation genes

Production of solvents was first detected between 4.5-6.5 h after the start of fermentation, which corresponded to the late exponential growth phase. The initiation of sporulation in *C. beijerinckii *8052 was concurrent with the onset of solventogenesis.

In *B. subtilis*, the gene *spo0H *encodes the earliest acting sigma factor associated with sporulation, σ^H^, which regulates the early sporulation genes, including *spo0A *and *sigF *operon [33]. The phosphorylated Spo0A (Spo0A ~ P) by phosphorelay system indirectly induces *spo0H *transcription by repressing *abrB *transcription [34]. In clostridia, no similar phosphorelay components and regulation system have been observed. It have been reported that in *C. acetobutylicum, spo0H *and *spo0A *were both constitutively expressed at constant levels throughout the growth cycle, and the amount of *spo0H *transcript was much lower than that of *spo0A *[34]. While a recent time-course transcriptional analysis of *C. acetobutylicum *indicated that *spo0A *was induced at the onset of sporulation and kept at high levels throughout the stationary phase [5]. In this study, the expression of *spo0H *(Cbei_0135) was constant from time points A-C, but was down-regulated 5.4-fold later during the fermentation (Figure 4B). The expression of *spo0A *(Cbei_1712) was induced early during the acidogenesis phase, and elevated by 2.2-fold during the transition phase and the onset of solventogenesis (time points B and C), after which the expression was slightly down-regulated (1.7-fold). Comparatively, the transcription level of *spo0A *was much higher than that of *spo0H *(Figure 4B). Putative σ^H^-dependent promoter and Spo0A-binding motif (0A box) have been identified upstream of the *spo0A *gene in *C. beijerinckii *8052 [35].

It is well established that *spo0A *is the master regulator for sporulation events in both bacilli and clostridia [5,34]. Phosphorylated *spo0A *has been reported to induce various targets, including the solventogenic operon and multiple sporulation sigma factor genes in *C. acetobutylicum *[3,36,37]. 0A boxes were also identified upstream of the operons related to chemotaxis/motility, suggesting the possible negative regulation of Spo0A on the expression of chemotaxis/motility genes [37-39]. A previous study involving insertional inactivation of *spo0A *indicated that Spo0A does also control the formation of solvents, spores and granulose in *C. beijerinckii *[40]. A recently developed *spo0A *mutant of *C. beijerinckii *8052 exhibited an asporogenous and non-septating phenotype [41]

The *sigF *operon (*spoIIAA*-*spoIIAB*-*sigF*) encodes the anti-anti-sigma factor (SpoIIAA), the anti-sigma factor (SpoIIAB) and the sigma factor σ^F^. In the "crisscross regulation" system of *B. subtilis*, both σ^H ^and Spo0A ~ P are required for the expression of the genes in this operon [42]. In this study, the *sigF *operon genes were induced before the onset of sporulation (time point B), and kept coordinated and high-regulated throughout the fermentation (Figure 4B).

SpoIIE is a phosphatase required for regulating the dephosphorylation of SpoIIAA, which helps to release the σ^F ^from SpoIIAB by forming an ADP-SpoIIAB-SpoIIAA complex [43]. In *B. subtilis*, expression of *spoIIE *starts about 1 h after the onset of sporulation, and peaks approximately 1 h later [44]. In the present study, high expression of *spoIIE *(Cbei_0097) in *C. beijerinckii *8052 was induced at time point C (51.3-fold up-regulated) following the onset of solvent production, and then quickly decreased to a lower level (but is still 7-fold higher than that at time point A) (Figure 4B).

σ^E ^is the first mother cell-specific sigma factor in *B. subtilis*, and the expression of *sigE *begins about 2 h after the onset of sporulation [45]. In *C. acetobutylicum*, the expression of *sigE *operon (*spoIIGA*-*sigE*, CAC1694 and CAC1695) does not exceed 1.3-fold higher than that at the initiation of fermentation [5]. In the case of *C. beijerinckii*, the expression of *sigE *operon (*spoIIGA*-*sigE*, Cbei_1119 and Cbei_1120) was up-regulated around 20-fold at time point C, and then decreased by 3- to 6-fold during late stationary phase (Figure 4B).

σ^G ^is a forespore-specific sigma factor and it is active after complete engulfment of the forespore by the mother cell [46]. In *B. subtilis*, significant expression of *sigG *begins 150 to 210 min after the onset of sporulation [47]. In the experiment presented here, the expression of *sigG *was up-regulated by 28.3-fold at time point C, and kept at high levels throughout the stationary process. More than 80 genes in *B. subtilis *which are related to spore protection and maturation were identified to be regulated by σ^G ^[48,49]. Among them, *spoIVB *is the gene encoding a signal protein for activating the later-acting sigma factor σ^K ^in the mother cell [50]; *spoVA *operon (*spoVAA*-*AE*) is a cluster of sporulation genes downstream of the *sigF *operon associated with dipicolinic acid transport into the developing spores [51,52]. In many clostridium species, *spoVAC, AD, AE *were found at the same locus, but *spoVAA *and *AB *are absent [53]. This is also the case for *C. beijerinckii *8052, and *spoVAC, AD, AE *(Cbei_815-817) were found to be organized in a single operon based on RNA-Seq data [19]. The expression of *spoIVB *and the *spoVA *operon was induced after the onset of high expression of *sigG*, and peaked at time points D and E (around 4- to 16-fold higher than that at time point A). The expression profiles of *sigG *and the putative σ^G^-regulated genes well confirmed their regulation manner in *C. beijerinckii *(Figure 4B).

Sporulation regulation factors genes *sigE *and *sigG *in *C. beijerinckii *in the present study were induced and up-regulated > 20-fold right after the onset of sporulation. While in *C. acetobutylicum*, no active expression of *sigE *and *sigG *was detected up to 7.5 h after the onset of sporulation [5]. This dissimilarity coincides with the fact that forespores and endspores develop more rapidly in *C. beijerinckii *8052 than in *C. acetobutylicum *824 [5,10].

In *B. subtilis, sigK *gene is expressed from a σ^E^-dependent promoter, and regulated by another transcription factor *spoIIID *[54-56]. The *sigK *gene is first expressed as an inactive pro-σ^k ^factor, and then cleaved to activate by a membrane-localized protease, SpoIVFB, whose activity is induced by SpoIVB, the signaling protein produced in the forespore [57,58]. The *sigK *gene in most clostridial species was also found to encode a pro-σ^k ^factor, and *spoIVFB *and *spoIVB *were identified in most clostridial species [53]. The product of Cbei_0501 (annotated as peptidase M50) in *C. beijerinckii *exhibits 36% amino acid identity (99 out of 273) and 62% positive (169 out of 273) to SpoIVFB in *C. acetobutylicum *by BLASTP. The expression of *spoIIID *(Cbei_0424) and *sigK *(Cbei_1115) was induced unexpectedly early at time point B, and increased up to 5.6- and 24.1-fold respectively at time point D (compared to time point B). Afterwards, both genes maintained at high expression levels. Nevertheless, significant expression of Cbei_0501 (putative *spoIVFB*) was only observed at time point C, and then the expression decreased to a lower level (Figure 4B).

In *B. subtilis, abrB *encodes a transition state regulator, whose transcription depression by Spo0A leads to a burst of σ^H ^synthesis at the initiation of sporulation [59]. Maximal expression of *abrB *in *B. subtilis *was observed 2 h before the onset of sporulation [60]. In *C. acetobutylicum*, three *abrB *homologs (CAC0310, 1941 and 3647) were identified [61], among which CAC0310 was suggested as the putative true transitional-state regulator [62]. However, the decrease of expression of CAC0310 was not observed at initiation of sporulation but the highest expression was observed after the onset of sporulation during a time course transcriptional analysis for the wild type *C. acetobutylicum *824 [5]. In *C. beijerinckii *8052, six loci were annotated as genes encoding AbrB family transcriptional regulator. Among them, expression of three (Cbei_2219, Cbei_2270 and Cbei_3199) are not detectable most of the time. Expression of Cbei_0088 decreased temporally by 10.8-fold at time point B, but up-regulated back to even higher levels afterwards. The expression of Cbei_3375 was significantly depressed at time point C (10.5-fold from point B), and kept at low levels during the stationary phase. Although Cbei_4885 was down-regulated by 3.2-fold at time point C, following expression was kept at relatively high levels and increased to a peak level at time point F (Figure 4B). In summary, none of the *abrB *genes in *C. beijerinckii *8052 displayed exact antagonistic expression pattern to that of *spo0H*. Whether there are *abrB *genes that play the similar role as *abrB *in *B. subtilis *warrants further study in respect that *spo0H *in this study had a very different transcriptional profile from that in *B. subtilis*.

As observed in other clostridium species, there was no orthologous phosphorelay system or sensory kinases in *C. beijerinckii *similar to those found in *B. subtilis *(KinA-KinE) for phosphorylating *spo0A *[53,63]. In *C. acetobutylicum*, it was suggested that the gene CAC3319 might act in a similar fashion to that of the sensory kinase genes in *B. subtilis *[4,37]. A recent study revealed evidence for two possible pathways for Spo0A activation in *C. acetobutylicum*, one dependent on CAC0323, and the other dependent on CAC0903 and CAC3319 [64]. In this study, with the time course transcriptional data, putative candidate proteins can be identified in C. *beijerinckii *that may play roles similar to sensory kinases for sporulation. Out of all the genes annotated as encoding sensory histidine kinases, ten were observed to be momentarily upregulated during the onset of sporulation (Figure 4B). Further molecular biology work focusing on specific genes needs to be carried out to define the genes that play the key roles in fermentation regulation.

#### Cell motility genes

The cell motility-related genes encode products responsible for chemotactic responses and flagellar assembly [65]. From the sequencing data, it was confirmed that a similar flagellar/chemotaxis multi-gene cluster exists in *C. beijerinckii *8052 (Cbei_4312-4302) as that in *C. acetobutylicum *(CAC2225-2215) [19,66]. Motility-related genes in clostridia and bacilli are usually down-regulated in sporulation cells [39]. In this study, most genes in the flagellar/chemotaxis cluster were down-regulated by 2- to 4-fold at the onset of sporulation and were kept at lower expression levels thereafter. However, the last three genes at the end of the cluster (Cbei_4304-4302, encoding cheW, fliM and fliY, respectively) were initially down-regulated by 2- to 3-fold at the onset of sporulation, and then unexpectedly increased during late stationary phase (Figure 4C).

There are several other genes in *C. beijerinckii *8052 annotated as motility-related. Among them, Cbei_0755 (*cheW*) and Cbei_4958 (*cheC*) were active during exponential phase, and were down-regulated by 4.1- and 3.1-fold respectively after cells entered the stationary phase (time point D). Another *cheW *gene (Cbei_3954), on the other hand, exhibited an unexpected antagonistic pattern, which was strongly expressed throughout the entire stationary phase and peaked at time point F (Figure 4C). The other chemotaxis genes were not active over the course of the fermentation process. The flagellar motility related genes *flgB *(Cbei_4271) and *flgC *(Cbei_4270) were strongly expressed during exponential phase (time points A and B), and down-regulated by 5.9- to 7.1-fold during the stationary phase. It has been reported that the expression of the flagellar/motility operon is negatively regulated by Spo0A in *B subtilis *[38].

#### Quorum sensing gene

The gene *luxS *(Cbei_1237) encodes S-ribosylhomocysteinase, which produces autoinducers to function in the Luxs/AI-2 quorum sensing mechanism [67]. Maximal autoinducer activity was observed during mid-exponential phase in *C. perfringens *with autoluminesence assays [67], and the expression of *luxS *in *C. acetobutylicum *was found to be roughly proportional to cell density in a time-course transcriptional analysis of *C. acetobutylicum *824 [5]. Sequence alignment of the protein encoded by the putative *luxS *gene in *C. beijerinckii *(Cbei_1237, 480 bp) and that in *C. acetobutylicum *(CAC2942, 477 bp) with BLASTP indicated that, out of 158 amino acids, there exists 74% identity (117 amino acids) and 84% positive (133 amino acids) matches. The expression of Cbei_1237 started at a high level during the acidogenesis phase, and then increased by 2.2-fold and peaked during the transition to solventogenesis (time point B), after which the expression decreased gradually to lower levels (Figure 4C).

#### Sugar transport genes

The phosphoenolpyruvate-dependent phosphotransferase system (PTS) is the predominant sugar uptake pathway in *C. beijerinckii *[68]. The first two PTS domains (general PTS proteins), referred as EI (enzyme I) and HPr (heat stable, histidine-phosphorylatable protein), phosphorylate the sugar in the cytoplasm, which has been transported across the cell membrane from the extracellular side by substrate-specific enzyme II (EII) domains. Phosphorylated sugar is then able to enter the glycolytic pathway [68]. In *C. beijerinckii*, EI and HPr are encoded by Cbei_0196 (*ptsI*) and Cbei_1219 (*ptsH*) respectively. The expression of both genes was high throughout the fermentation process with a 2- to 3-fold decrease after onset of solventogenesis (Figure 4C). This agrees well with the previous finding that ATP-dependent glucose phosphorylation was predominant and phosphoenolpyruvate-dependent glucose phosphorylation was repressed during solventogenic stage for both *C. beijerinckii *8052 and its mutant *C. beijerinckii *BA101 strains, although the latter exhibited a nearly 2-fold-greater ATP-dependent phosphorylation rate during solventogenic stage [69].

Previous competition studies indicated that glucose and mannose were assimilated by the same PTS in *C. beijerinckii *[69,70]. The mannose family PTS transporters are commonly found in gammaproteobacteria and firmicutes, including *C. acetobutylicum *and *C. beijerinckii*, and exhibit broad substrate specificity for glucose, mannose, sorbose, fructose, and a variety of other sugars [10,71]. In the gene cluster encoding for the mannose family PTS transporters, *manIIAB *encodes a membrane fusion protein of the IIA and IIB subunits involved in sugar phosphorylation, *manIIC *encodes a substrate-specific permease, and *manIID *encodes a mannose family-specific auxiliary protein (essential but of unknown function) [71]. The RNA-Seq data revealed that these three genes were organized in the same operon (Cbei_0711-0713) in *C. beijerinckii *8052 [19], and the expression of *manIIAB *(Cbei_0711), *manIIC *(Cbei_0712) and *manIID *(Cbei_0713) was highly coordinated and unexpectedly up-regulated by 11.6, 20.4 and 31.6-fold respectively after onset of solventogenesis (Figure 4C).

There are 13 PTS domains identified in *C. acetobutylicum*, out of which six belongs to the glucose-glucoside (Glc) family [72]. Homologs were identified in *C. beijerinckii *for all the gene clusters encoding the Glc family PTS transporters in *C. acetobutylicum*. Among them, only Cbei_0751, Cbei_3273, Cbei_4532, Cbei_4533 and Cbei_4705 were actively expressed during the fermentation process, and Cbei_4533 (encoding N-acetylglucosamine-specific IIA subunit) and Cbei_4532 (encoding N-acetylglucosamine-specific IIBC subunit) were especially strongly expressed throughout the batch fermentation process (Figure 4C). By comparing the expression dynamics of gene clusters encoding for the Glc family PTS transporters and those for the mannose family PTS transporters, it could be inferred that the Glc family PTS transporters (especially those encoded by Cbei_4532 and _4533) play important roles during exponential and early stationary phases, while the mannose family PTS transporters may be more closely related to stationary and sporulation events.

#### Self-organizing maps (SOM) analysis of functional groups

**Figure 5:**
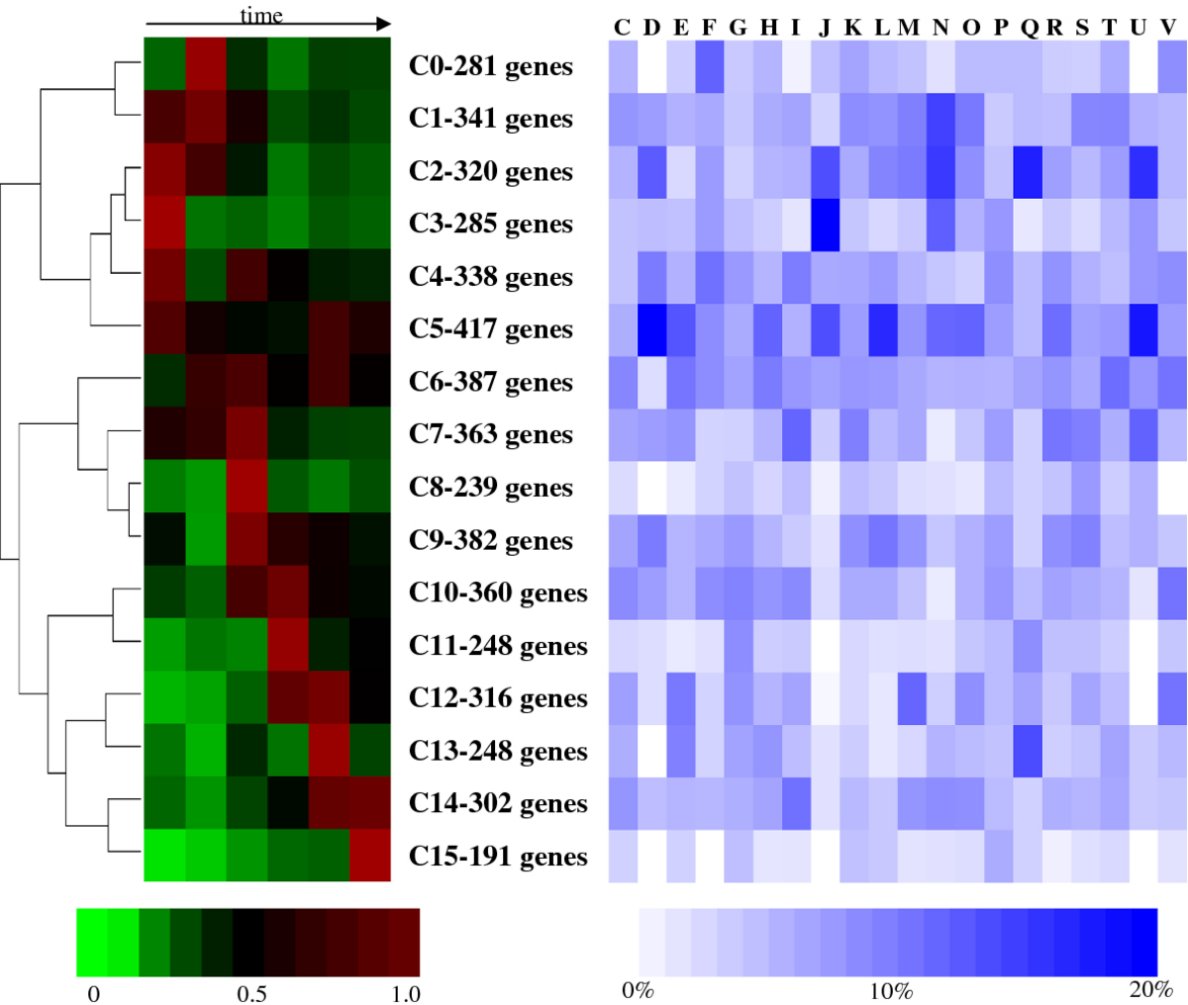
**SOM clusters of gene expression profiles during the batch fermentation**. SOM clusters are grouped by average-linkage hierarchical clustering based on expression pattern similarities. Distribution of COG categories across the clusters is represented colorimetrically on the right. COG functional groups are from the *C. beijerinckii *8052 genome annotation defined by the COG database [73].

SOM analysis was employed to investigate the global expression patterns, especially for genes belonging to specific COG categories across the genome (Figure 5) [5]. The expression of genes in clusters C2 and C3 was up-regulated during early exponential-growth phase and decreased to lower levels afterwards. While these two clusters jointly contain 605 genes (12.1%, out of totally 5018 in the analysis), they are enriched with 49.2% of the genes related to translation (COG J), and 28.1% of genes related to cell motility (COG N). Cell motility genes were also over-represented by 14.8% in cluster C1, where the expression levels of genes were lower in the stationary phase than that in the exponential phase.

#### Carbohydrate transport and metabolism (COG G)

Nine of the glycolysis genes discussed above (Figure 4A) falls into clusters C1 and C2, indicating more active glycolytic activities during the exponential-growth phase. On the other hand, solventogenic clostridia initiate granulose production at the early stationary phase; Granulose is a glycogen-like polymer used by bacteria for energy storage under severe environments [74]. Operon prediction for *C. acetobutylicum *indicated that *glgC *(CAC2237, encoding glucose-1-phosphate adenylyltransferase), *glgD *(CAC2238, encoding ADP-glucose pyrophosphorylase), *glgA *(CAC2239, encoding glycogen synthase) and CAC2240 (encoding cyclomaltodextrin glucanotransferase domain-containing protein) express as a single transcript [66]. In addition, *glgP *encodes a granulose phosphorylase (CAC1664, functions to depolymerize granulose), and *csrA *(CAC2209) encodes a putative carbon storage regulator, which was reported to repress the expression of both *glgA *and *glgP *in *Escherichia coli *[5,75]. In *C. beijerinckii *8052, it was observed that *glgC *(Cbei_4905) and *glgD *(Cbei_4904) were organized in a operon, while the gene encoding 1,4-alpha-glucan branching enzyme (Cbei_4909), *glgA *(Cbei_4908), *glgP *(Cbei_4907) and the gene encoding the catalytic region of alpha amylase (Cbei_4906) were organized in another adjacent operon [19]. Cbei_4295 was annotated as the *csrA *gene. The five putative granulose formation genes (Cbei_4905-4909) in *C. beijerinckii *8052 were grouped into cluster C6 based on their expression profiles (Figure 5). Expression of most granulose formation genes were up-regulated by 2- to 4-fold at transition phase from acidogenesis to solventogenesis (time point B) and maintained at high expression levels over the stationary phase (point C-E) (Figure 4C). Notwithstanding, the expression of *csrA *(Cbei_4295) was only detectable at A, C and F, which was much lower than those of the other granulose formation genes (Figure 4C). It is not yet clear whether the *csrA *gene in *C. beijerinckii *functions similarly to that in *E. coli*.

#### Translation genes (COG J)

There are 181 translation-related genes included in the SOM analysis. The majority were grouped in the clusters where the gene expression is active during exponential phase (78% enriched in C1-C5). There are 58 genes in *C. beijerinckii *genome encoding ribosomal proteins. Out of the 56 genes encoding ribosomal proteins included in the SOM analysis, 46 were clustered in C3, indicating that the protein synthesis machinery in the cell is mostly active during the early exponential phase (Figure 5). Similar results were reported for *C. acetobutylicum*, where most of the ribosomal protein genes were down-regulated at least 3-fold in stationary phase [5].

#### Signal transduction mechanisms genes (COG T)

There are 371 genes (around 7.4% of all genes) annotated as signal transduction related genes in *C. beijerinckii*. SOM analysis did not reveal any specific enriched clusters for these genes. However, they are slightly over-represented in cluster C0 and C1 (jointly 16% of category T), where the expression levels are higher during the exponential phase (Figure 5). Among them, there are important cell chemotaxis and motility associated sensory transduction genes (Cbei_0279, _0665, _0755, _0804, _4012, _4161, _4307, _4309, _4310, _4466, _4604 and _4832). The expression of genes in clusters C8-C10 was momentarily up-regulated at onset of stationary phase and decreased quickly afterwards (Figure 5). The signal transduction genes in clusters C8-C10 may represent important signal transducers for solventogenesis and sporulation events. Among them, there are genes encoding response regulator receiver proteins, integral membrane sensor signal transduction histidine kinase and two-component transcriptional regulators (Additional file 4: Table S4) [76,77].

#### Correlations of RNA-Seq and qRT-PCR

The expression measurement with RNA-Seq approach was validated using qRT-PCR for selected genes. For all the selected genes, the dynamic gene expression profiles over time measured with RNA-Seq have very similar patterns as those measured with qRT-PCR (Additional file 5: Figure S1). We previously showed that good correlation exists between the overall gene expression quantification with RNA-Seq and that obtained with microarray or qRT-PCR [19]. These results demonstrate that RNA-Seq is an effective approach for quantification of gene expression.

Previously, Shi and Blaschek reported the transcriptional analysis of *C. beijerinckii *using a ca. 500-gene set DNA microarray where only the expression of genes orthologous to those previously found to be important to the physiology of *C. acetobutylicum *824 was examined [10]. In this study, employing RNA-Seq technology, for the first time the genome-wide dynamic transcriptional profiling of *C. beijerinckii *correlated with the physiological activities over the course of fermentation process has been revealed with a much larger dynamic range. In addition, single-nucleotide resolution RNA-Seq data also revealed the transcriptome structural organization of *C. beijerinckii *8052 in depth [19]. The transcriptome structural information together with the genome-wide dynamic transcriptional profiling can provide essential references for further improvement of *C. beijerinckii *strains by employing system biology-based strategies and metabolic engineering approaches. Furthermore, the genome-wide RNA-Seq analysis also disclosed the different expression dynamics of many allelic genes in the genome of *C. beijerinckii *8052, which provided important insight for future studies on the properties of potential isoenzymes and their associated functions during specific fermentation stages.

## Conclusions

By employing single-base resolution RNA-Seq technology, the genome-wide transcriptional dynamics of *C. beijerinckii *8052 throughout the course of a batch fermentation was revealed in great depth. RNA-Seq technology can not only quantify the differential transcription of specific genes over time, but is also able to compare transcription levels of different genes in the same sample. Overall dynamic transcription profiles demonstrated obvious temporal variation trend throughout the fermentation, corresponding to the physiological state change of the cells. Glycolysis genes demonstrated higher expression during acidogenesis phase. The expression of acid formation genes declined at the onset of solvent formation, in accordance with the metabolic pathway shift from acidogenesis to solventogenesis. The *sol *operon, including *adc *as a part, was up-regulated at the onset of solventogenesis and maintained at high levels through the stationary phase. Most sporulation genes in *C. beijerinckii *8052 demonstrated similar temporal expression patterns to those observed in *B. subtilis *and *C. acetobutylicum*, while sporulation sigma factor genes *sigE *and *sigG *exhibited accelerated and stronger expression in *C. beijerinckii *8052, consistent with the more rapid forespore and endspore development in this strain. The PTS transport system encoding genes were highly expressed during acidogenesis and declined 2- to 3-fold after entering solventogenesis phase, agreeing well with the report that ATP-dependent glucose phosphorylation was predominant and phosphoenolpyruvate-dependent PTS was repressed during solventogenic stage for *C. beijerinckii*. Finally, SOM and clustering analysis revealed that genes of specific COG functional classes demonstrated global gene expression patterns corresponding to the cell physiological change and fermentation pathway switch.

## Methods

### Bacterial culture and fermentation experiment

Laboratory stocks of *C. beijerinckii *8052 spores were stored in sterile H_2_O at 4°C [78]. Spores were heat-shocked at 80°C for 10 min, followed by cooling on ice for 5 min. The heat-shocked spores were inoculated at a 1% inoculum level into tryptone-glucose-yeast extract (TGY) medium containing 30 g L^-1 ^tryptone, 20 g L^-1 ^glucose, 10 g L^-1 ^yeast extract and 1 g L^-1 ^L-cysteine. The TGY culture was incubated at 35 ± 1°C for 12-14 h in an anaerobic chamber under N_2_:CO_2_:H_2 _(volume ratio of 85:10:5) atmosphere. Subsequently, actively growing culture was inoculated into a model solution containing 60 g L^-1 ^glucose, 1 g L^-1 ^yeast extract, and filter-sterilized P2 medium [79] in a Sixfors bioreactor system (Infors AG, Bottmingen, Switzerland). Oxygen-free nitrogen was flushed through the broth to initiate anaerobiosis until the culture initiated its own gas production (CO_2 _and H_2_). Temperature was controlled at 35 ± 1°C. A stirring at 50 rpm was employed for mixing. Cell density and product concentration were monitored through the course of fermentation. For sequencing purpose, samples were taken over the early exponential, late exponential and stationary phases (samples A-F at 2, 4.5, 10, 14, 17 and 26.5 h respectively as shown in Figure 1A).

### Culture growth and fermentation products analysis

Culture growth was measured by following optical density (OD) in the fermentation broth at A_600 _using a BioMate 5 UV-vis Spectrophotometer (Thermo Fisher Scientific Inc., Waltham, MA). ABE, acetic acid, and butyric acid concentrations were quantified using gas chromatography (GC) system as previously described [79].

### RNA isolation, library construction and sequencing

For RNA isolation, 10 ml cultures were harvested at each time point, and centrifuged at 4,000 × g for 10 min at 4°C. Total RNA was extracted from the cell pellet using Trizol reagent based on manufacture's protocol (Invitrogen, Carlsbad, CA) and further purified using RNeasy mini kit (Qiagen, Valencia, CA). DNA was removed using a DNA-*free*™ kit (Ambion Inc., Austin, TX). RNA quality was assessed using a 2100 bioanalyzer (Agilent Technologies, Santa Clara, CA). RNA concentration was determined with a nanodrop (Biotek Instruments, Winooski, VT). Bacterial 16S and 23S ribosomal RNAs were removed with a MICROBExpress™ kit (Ambion Inc., Austin, TX). The enriched mRNA was converted to a RNA-Seq library using the mRNA-Seq library construction kit (Illumina Inc., San Diego, CA) following manufacturer's protocols. For samples A to F, two samples were pooled and sequenced on one single lane of an eight-lane flow cell with the Genome Analyzer IIx system (Illumina Inc., San Diego, CA). However, sample F yielded a poor read quality following the first sequencing. In order to obtain enough sequencing depth, sample F was sequenced again using one single lane under otherwise identical conditions. The derived sequence reads were 75 nt long. The overall error rate of the control DNA was < 0.6%. The total number of reads generated from each library is summarized in Table 1.

### RNA-Seq data analysis

The generated 75-nt reads were mapped to the *C. beijerinckii *8052 genome using MAQ, and those that did not align uniquely to the genome were discarded [80]. The quality parameter (-q) used in MAQ pileup was set to 30. Each base was assigned a value based on the number of mapped sequence coverage. The quantitative gene expression value, RPKM (reads/Kb/Million), was calculated using custom Perl scripts by normalizing the sequence coverage over the gene length and total unambiguously mapped reads in each library [13,18].

Z score was calculated as Z = (X-X_av_)/X_SD_. X is the log_2_-transformed RPKM value for a given gene in a specific sample, X_av _is the mean of log_2_-transformed RPKM values for that gene in all six samples, and X_SD _is the standard deviation of log_2_-transformed RPKM for that gene across all samples [18,21].

The gene expression results were visualized colorimetrically using heatmap plots. R language and the open source software Bioconductor based on R were used for the data analysis and plots [81-83]. Specifically, functions in Heatplus package in Bioconductor and the R package gplots were employed for constructing the heatmap plots. Time course RPKM values were first transformed to log_2_-scale. The log_2_-transfromed RPKM values were then properly centered for better representation of the data by the heatmap plots. More details about this method were summarized in the Additional file 6.

### Self-organizing maps (SOM) analysis

Global gene expression patterns were analyzed with self-organizing maps (SOM) analysis [5]. The RPKM value of a gene at each time point was first divided by the square root of the sum of the squares of the gene's RPKM values at all six time points, and therefore the sum of the squares of each gene's standardized ratios within the experiment is 1. The preprocessed gene expression values were then standardized and grouped into 16 SOM clusters based on the expression pattern similarity (Figure 5). In order to examine the SOM clusters for enriched Clusters of Orthologous Groups of proteins (COG), the frequency at which the genes in each cluster fell in every COG category was calculated and plotted colorimetrically (Figure 5). If one gene belongs to more than one COG categories, the gene was counted once for each category [5]. Data were processed and visualized with Gene Cluster 3.0 [84] and the SOMClustering and Java TreeView packages in GenePattern [85].

### Real time qRT-PCR

Quantitative reverse transcription PCR (qRT-PCR) was performed for selected genes in order to validate the quantification of gene expression levels obtained by RNA-Seq. Nine genes that are important involved in the central pathways for glycolysis, acidogenesis and solventogenesis were chosen for the examination. Cbei_2428 was used as the endogenous control gene based on its relatively constant expression levels throughout the fermentation process as discussed earlier. Specific primer sequences for tested genes are listed in Additional file 7: Table S5. Triplicate reactions were performed using Power SYBR green PCR master mix (Applied Biosystems, Carlsbad, CA) on an ABI Prism 7900HT fast real-time PCR machine (Applied Biosystems). The normalized threshold value (ΔCt) of each gene along time course was compared to that of the same gene at the first time point (time point A in Figure 1A).

### RNA-Seq data accession number

The RNA-Seq sequencing data have been deposited in the NCBI Sequence Read Archive (SRA) under the accession number SRA045799.

## Competing interests

The authors declare that they have no competing interests.

## Authors' contributions

YW, XL, HPB conceived and designed the study. YW and XL performed the experiments. YW, XL and YM analyzed the RNA-Seq data. YW, XL and HPB wrote the manuscript, with input from all authors. All authors discussed the results, read and approved the final manuscript.

## Supplementary Material

Additional file 1 **The genes with Z-score > 1.7 in each sample**. Click here for file

Additional file 2 **The top 100 highest expressed genes in samples B and D**. Click here for file

Additional file 3 **The expression of the putative alcohol dehydrogenase genes in *C. beijerinckii *8052**. Click here for file

Additional file 4 **The putative signal transduction genes in SOM clusters C8-C10 in *C. beijerinckii *8052**. Click here for file

Additional file 5 **The qRT-PCR verification of RNA-Seq quantification results for selected genes**. The fold change of each gene's expression along time course measured by either qRT- PCR (in black) or RNA-Seq (in red) was compared to that of the same gene at first time point (time point A in Figure 1A). (A) Cbei_4852, *pfk*. (B) Cbei_1903, *fba*. (C) Cbei_4851, *pyk*. (D) Cbei_0203, *ptb*. (E) Cbei_0204, *buk*. (F) Cbei_3833, *ctfA*. (G) Cbei_3834, *ctfB*. (H) Cbei_0411, *thlA*. (I) Cbei_0325, *hbd*. Click here for file

Additional file 6 **RNA-Seq data analysis**. Click here for file

Additional file 7 **Genes and primer sequences for qRT-PCR test**. Click here for file

## References

[B1] Dürre P Biobutanol: an attractive biofuel Biotechnol J 2007 2 12 1525 1534 10.1002/biot.200700168 17924389

[B2] Ezeji T Blaschek HP Fermentation of dried distillers' grains and solubles (DDGS) hydrolysates to solvents and value-added products by solventogenic clostridia Bioresour Technol 2008 99 12 5232 5242 10.1016/j.biortech.2007.09.032 17967532

[B3] Alsaker KV Spitzer TR Papoutsakis ET Transcriptional analysis of *spo0A *overexpression in *Clostridium acetobutylicum *and its effect on the cell's response to butanol stress J Bacteriol 2004 186 7 1959 1971 10.1128/JB.186.7.1959-1971.2004 15028679 PMC374416

[B4] Tomas CA Beamish J Papoutsakis ET Transcriptional analysis of butanol stress and tolerance in *Clostridium acetobutylicum* J Bacteriol 2004 186 7 2006 2018 10.1128/JB.186.7.2006-2018.2004 15028684 PMC374415

[B5] Alsaker KV Papoutsakis ET Transcriptional program of early sporulation and stationary-phase events in *Clostridium acetobutylicum* J Bacteriol 2005 187 20 7103 7118 10.1128/JB.187.20.7103-7118.2005 16199581 PMC1251621

[B6] Alsaker KV Paredes CJ Papoutsakis ET Design, optimization and validation of genomic DNA microarrays for examining the *Clostridium acetobutylicum *transcriptome Biotechnol Bioprocess Eng 2005 10 5 432 443 10.1007/BF02989826

[B7] Jones SW Paredes CJ Tracy B Cheng N Sillers R Senger RS Papoutsakis ET The transcriptional program underlying the physiology of clostridial sporulation Genome Biol 2008 9 7 R114 10.1186/gb-2008-9-7-r114 18631379 PMC2530871

[B8] Alsaker KV Paredes C Papoutsakis ET Metabolite stress and tolerance in the production of biofuels and chemicals: gene-expression-based systems analysis of butanol, butyrate, and acetate stresses in the anaerobe *Clostridium acetobutylicum* Biotechnol Bioeng 2010 105 6 1131 1147 19998280 10.1002/bit.22628

[B9] Janssen H Doring C Ehrenreich A Voigt B Hecker M Bahl H Fischer RJ A proteomic and transcriptional view of acidogenic and solventogenic steady-state cells of *Clostridium acetobutylicum *in a chemostat culture Appl Microbiol Biotechnol 2010 87 6 2209 2226 10.1007/s00253-010-2741-x 20617312 PMC3227527

[B10] Shi Z Blaschek HP Transcriptional analysis of *Clostridium beijerinckii *NCIMB 8052 and the hyper-butanol-producing mutant BA101 during the shift from acidogenesis to solventogenesis Appl Environ Microbiol 2008 74 24 7709 7714 10.1128/AEM.01948-08 18849451 PMC2607153

[B11] Passalacqua KD Varadarajan A Ondov BD Okou DT Zwick ME Bergman NH Structure and complexity of a bacterial transcriptome J Bacteriol 2009 191 10 3203 3211 10.1128/JB.00122-09 19304856 PMC2687165

[B12] Perkins TT Kingsley RA Fookes MC Gardner PP James KD Yu L Assefa SA He M Croucher NJ Pickard DJ A strand-specific RNA-Seq analysis of the transcriptome of the typhoid bacillus *Salmonella typhi* PLoS Genet 2009 5 7 e1000569 10.1371/journal.pgen.1000569 19609351 PMC2704369

[B13] Mortazavi A Williams BA McCue K Schaeffer L Wold B Mapping and quantifying mammalian transcriptomes by RNA-Seq Nat Methods 2008 5 7 621 628 10.1038/nmeth.1226 18516045 PMC13303166

[B14] Nagalakshmi U Wang Z Waern K Shou C Raha D Gerstein M Snyder M The transcriptional landscape of the yeast genome defined by RNA sequencing Science 2008 320 1344 1349 10.1126/science.1158441 18451266 PMC2951732

[B15] van Vliet AHM Next generation sequencing of microbial transcriptomes: challenges and opportunities FEMS Microbiol Lett 2010 302 1 1 7 10.1111/j.1574-6968.2009.01767.x 19735299

[B16] Wang Z Gerstein M Snyder M RNA-Seq: a revolutionary tool for transcriptomics Nat Rev Genet 2009 10 1 57 63 10.1038/nrg2484 19015660 PMC2949280

[B17] Legendre M Audic S Poirot O Hingamp P Seltzer V Byrne D Lartigue A Lescot M Bernadac A Poulain J mRNA deep sequencing reveals 75 new genes and a complex transcriptional landscape in Mimivirus Genome Res 2010 20 5 664 674 10.1101/gr.102582.109 20360389 PMC2860168

[B18] Severin A Woody J Bolon Y-T Joseph B Diers B Farmer A Muehlbauer G Nelson R Grant D Specht J RNA-Seq Atlas of *Glycine max*: a guide to the soybean transcriptome BMC Plant Biol 2010 10 1 160 10.1186/1471-2229-10-160 20687943 PMC3017786

[B19] Wang Y Li X Mao Y Blaschek H Single-nucleotide resolution analysis of the transcriptome structure of *Clostridium beijerinckii *NCIMB 8052 using RNA-Seq BMC Genomics 2011 12 479 10.1186/1471-2164-12-479 21962126 PMC3271303

[B20] Hill MO Gauch HG Detrended correspondence analysis: an improved ordination technique Vegetatio 1980 42 1-3 47 58 10.1007/BF00048870

[B21] Benedito VA Torres-Jerez I Murray JD Andriankaja A Allen S Kakar K Wandrey M Verdier J Zuber H Ott T A gene expression atlas of the model legume *Medicago truncatula* Plant J 2008 55 3 504 513 10.1111/j.1365-313X.2008.03519.x 18410479

[B22] Winzer K Lorenz K Zickner B Dürre P Differential regulation of two thiolase genes from *Clostridium acetobutylicum *DSM 792 J Mol Microbiol Biotechnol 2000 2 4 531 541 11075929

[B23] Grimmler C Janssen H Krausse D Fischer RJ Bahl H Dürre P Liebl W Ehrenreich A Genome-wide gene expression analysis of the switch between acidogenesis and solventogenesis in continuous cultures of *Clostridium acetobutylicum* J Mol Microbiol Biotechnol 2011 20 1 1 15 10.1159/000320973 21212688

[B24] Cornillot E Nair RV Papoutsakis ET Soucaille P The genes for butanol and acetone formation in *Clostridium acetobutylicum *ATCC 824 reside on a large plasmid whose loss leads to degeneration of the strain J Bacteriol 1997 179 17 5442 5447 9286999 10.1128/jb.179.17.5442-5447.1997 PMC179415

[B25] Gerischer U Dürre P Cloning, sequencing, and molecular analysis of the acetoacetate decarboxylase gene region from *Clostridium acetobutylicum* J Bacteriol 1990 172 12 6907 6918 2254264 10.1128/jb.172.12.6907-6918.1990 PMC210810

[B26] Petersen DJ Cary JW Vanderleyden J Bennett GN Sequence and arrangement of genes encoding enzymes of the acetone-production pathway of *Clostridium acetobutylicum *ATCC 824 Gene 1993 123 1 93 97 10.1016/0378-1119(93)90545-E 8423010

[B27] Gerischer U Dürre P mRNA analysis of the *adc *gene region of *Clostridium acetobutylicum *during the shift to solventogenesis J Bacteriol 1992 174 2 426 433 1370288 10.1128/jb.174.2.426-433.1992 PMC205733

[B28] Feustel L Nakotte S Dürre P Characterization and development of two reporter gene systems for *Clostridium acetobutylicum* Appl Environ Microbiol 2004 70 2 798 803 10.1128/AEM.70.2.798-803.2004 14766557 PMC348925

[B29] Sauer U Dürre P Differential induction of genes related to solvent formation during the shift from acidogenesis to solventogenesis in continuous culture of *Clostridium acetobutylicum* FEMS Microbiol Lett 1995 125 1 115 120 10.1111/j.1574-6968.1995.tb07344.x

[B30] Chen J-S Alcohol dehydrogenase: multiplicity and relatedness in the solvent-producing clostridia FEMS Microbiol Rev 1995 17 3 263 273 10.1111/j.1574-6976.1995.tb00210.x 7576768

[B31] Walter KA Bennett GN Papoutsakis ET Molecular characterization of two *Clostridium acetobutylicum *ATCC 824 butanol dehydrogenase isozyme genes J Bacteriol 1992 174 22 7149 7158 1385386 10.1128/jb.174.22.7149-7158.1992 PMC207405

[B32] Welch RW Rudolph FB Papoutsakis ET Purification and characterization of the NADH-dependent butanol dehydrogenase from *Clostridium acetobutylicum *(ATCC 824) Arch Biochem Biophys 1989 273 2 309 318 10.1016/0003-9861(89)90489-X 2673038

[B33] Predich M Nair G Smith I *Bacillus subtilis *early sporulation genes *kinA, spo0A*, and *spo0F *are transcribed by the RNA polymerase containing σ^H^ J Bacteriol 1992 174 9 2771 2778 1569009 10.1128/jb.174.9.2771-2778.1992 PMC205927

[B34] Dürre P Hollergschwandner C Initiation of endospore formation in *Clostridium acetobutylicum* Anaerobe 2004 10 2 69 74 10.1016/j.anaerobe.2003.11.001 16701502

[B35] Wilkinson SR Young DI Gareth Morris J Young M Molecular genetics and the initiation of solventogenesis in *Clostridium beijerinckii *(formerly *Clostridium acetobutylicum*) NCIMB 8052 FEMS Microbiol Rev 1995 17 3 275 285 10.1111/j.1574-6976.1995.tb00211.x 7576769

[B36] Harris LM Welker NE Papoutsakis ET Northern, morphological, and fermentation analysis of *spo0A *inactivation and overexpression in *Clostridium acetobutylicum *ATCC 824 J Bacteriol 2002 184 13 3586 3597 10.1128/JB.184.13.3586-3597.2002 12057953 PMC135115

[B37] Tomas CA Alsaker KV Bonarius HPJ Hendriksen WT Yang H Beamish JA Paredes CJ Papoutsakis ET DNA array-based transcriptional analysis of asporogenous, nonsolventogenic *Clostridium acetobutylicum *strains SKO1 and M5 J Bacteriol 2003 185 15 4539 4547 10.1128/JB.185.15.4539-4547.2003 12867463 PMC165787

[B38] Molle V Fujita M Jensen ST Eichenberger P Gonzalez-Pastor JE Liu JS Losick R The Spo0A regulon of *Bacillus subtilis* Mol Microbiol 2003 50 5 1683 1701 10.1046/j.1365-2958.2003.03818.x 14651647

[B39] Paredes CJ Alsaker KV Papoutsakis ET A comparative genomic view of clostridial sporulation and physiology Nat Rev Microbiol 2005 3 12 969 978 10.1038/nrmicro1288 16261177

[B40] Ravagnani A Jennert KCB Steiner E Grunberg R Jefferies JR Wilkinson SR Young DI Tidswell EC Brown DP Youngman P Spo0A directly controls the switch from acid to solvent production in solvent-forming clostridia Mol Microbiol 2000 37 5 1172 1185 10.1046/j.1365-2958.2000.02071.x 10972834

[B41] Heap JT Kuehne SA Ehsaan M Cartman ST Cooksley CM Scott JC Minton NP The ClosTron: Mutagenesis in *Clostridium *refined and streamlined J Microbiol Methods 2010 80 1 49 55 10.1016/j.mimet.2009.10.018 19891996

[B42] Losick R Stragier P Crisscross regulation of cell-type-specific gene expression during development in *B. subtilis* Nature 1992 355 6361 601 604 10.1038/355601a0 1538747

[B43] Arigoni F Duncan L Alper S Losick R Stragier P SpoIIE governs the phosphorylation state of a protein regulating transcription factor σ^F ^during sporulation in *Bacillus subtilis* Proc Natl Acad Sci USA 1996 93 8 3238 3242 10.1073/pnas.93.8.3238 8622920 PMC39589

[B44] York K Kenney TJ Satola S Moran CP Jr Poth H Youngman P Spo0A controls the σ^A^-dependent activation of *Bacillus subtilis *sporulation-specific transcription unit *spoIIE* J Bacteriol 1992 174 8 2648 2658 1556084 10.1128/jb.174.8.2648-2658.1992 PMC205905

[B45] Kenney TJ Moran CP Jr Organization and regulation of an operon that encodes a sporulation-essential sigma factor in *Bacillus subtilis* J Bacteriol 1987 169 7 3329 3339 2439490 10.1128/jb.169.7.3329-3339.1987 PMC212387

[B46] Cutting S Panzer S Losick R Regulatory studies on the promoter for a gene governing synthesis and assembly of the spore coat in *Bacillus subtilis* J Mol Biol 1989 207 2 393 404 10.1016/0022-2836(89)90262-3 2474075

[B47] Sun DX Cabrera-Martinez RM Setlow P Control of transcription of the *Bacillus subtilis spoIIIG *gene, which codes for the forespore-specific transcription factor σ^G^ J Bacteriol 1991 173 9 2977 2984 1902213 10.1128/jb.173.9.2977-2984.1991 PMC207881

[B48] Steil L Serrano M Henriques AO Volker U Genome-wide analysis of temporally regulated and compartment-specific gene expression in sporulating cells of *Bacillus subtilis* Microbiology-(UK) 2005 151 399 420 10.1099/mic.0.27493-0 15699190

[B49] Wang ST Setlow B Conlon EM Lyon JL Imamura D Sato T Setlow P Losick R Eichenberger P The forespore line of gene expression in *Bacillus subtilis* J Mol Biol 2006 358 1 16 37 10.1016/j.jmb.2006.01.059 16497325

[B50] Cutting S Driks A Schmidt R Kunkel B Losick R Forespore-specific transcription of a gene in the signal transduction pathway that governs Pro-σ^K ^processing in *Bacillus subtilis* Genes Dev 1991 5 3 456 466 10.1101/gad.5.3.456 1900494

[B51] Kunst F Ogasawara N Moszer I Albertini AM Alloni G Azevedo V Bertero MG Bessieres P Bolotin A Borchert S The complete genome sequence of the Gram-positive bacterium *Bacillus subtilis* Nature 1997 390 6657 249 256 10.1038/36786 9384377

[B52] Tovar-Rojo F Chander M Setlow B Setlow P The products of the *spoVA *operon are involved in dipicolinic acid uptake into developing spores of *Bacillus subtilis* J Bacteriol 2002 184 2 584 587 10.1128/JB.184.2.584-587.2002 11751839 PMC139579

[B53] Stragier P Sonenshein A, Hoch J, Losick R A gene odyssey: exploring the genomes of endospore-forming bacteria Bacillus subtilis and its closest relatives: from genes to cells 2002 Washington, DC: ASM Press 519 525

[B54] Driks A Losick R Compartmentalized expression of a gene under the control of sporulation transcription factor σ^E ^in *Bacillus subtilis* Proc Natl Acad Sci USA 1991 88 22 9934 9938 10.1073/pnas.88.22.9934 1946462 PMC52841

[B55] Kunkel B Kroos L Poth H Youngman P Losick R Temporal and spatial control of the mother-cell regulatory gene *spoIIID *of *Bacillus subtilis* Genes Dev 1989 3 11 1735 1744 10.1101/gad.3.11.1735 2514119

[B56] Halberg R Kroos L Sporulation regulatory protein SpoIIID from *Bacillus subtilis *activates and represses transcription by both mother-cell-specific forms of RNA polymerase J Mol Biol 1994 243 3 425 436 10.1006/jmbi.1994.1670 7966271

[B57] Lu S Halberg R Kroos L Processing of the mother-cell σ factor, σ^K^, may depend on events occurring in the forespore during *Bacillus subtilis *development Proc Natl Acad Sci USA 1990 87 24 9722 9726 10.1073/pnas.87.24.9722 2124700 PMC55245

[B58] Green DH Cutting SM Membrane topology of the *Bacillus subtilis *Pro-σ^K ^processing complex J Bacteriol 2000 182 2 278 285 10.1128/JB.182.2.278-285.2000 10629171 PMC94274

[B59] Weir J Predich M Dubnau E Nair G Smith I Regulation of *spo0H*, a gene coding for the *Bacillus subtilis *σ^H ^factor J Bacteriol 1991 173 2 521 529 1898930 10.1128/jb.173.2.521-529.1991 PMC207041

[B60] Jiang M Shao WL Perego M Hoch JA Multiple histidine kinases regulate entry into stationary phase and sporulation in *Bacillus subtilis* Mol Microbiol 2000 38 3 535 542 10.1046/j.1365-2958.2000.02148.x 11069677

[B61] Nolling J Breton G Omelchenko MV Makarova KS Zeng QD Gibson R Lee HM Dubois J Qiu DY Hitti J Genome sequence and comparative analysis of the solvent-producing bacterium *Clostridium acetobutylicum* J Bacteriol 2001 183 16 4823 4838 10.1128/JB.183.16.4823-4838.2001 11466286 PMC99537

[B62] Scotcher MC Rudolph FB Bennett GN Expression of *abrB310 *and *sinR*, and effects of decreased *abrB310 *expression on the transition from acidogenesis to solventogenesis, in *Clostridium acetobutylicu *ATCC 824 Appl Environ Microbiol 2005 71 4 1987 1995 10.1128/AEM.71.4.1987-1995.2005 15812030 PMC1082569

[B63] Stephenson K Hoch JA Evolution of signalling in the sporulation phosphorelay Mol Microbiol 2002 46 2 297 304 10.1046/j.1365-2958.2002.03186.x 12406209

[B64] Steiner E Dago AE Young DI Heap JT Minton NP Hoch JA Young M Multiple orphan histidine kinases interact directly with Spo0A to control the initiation of endospore formation in *Clostridium acetobutylicum* Mol Microbiol 2011 80 3 641 654 10.1111/j.1365-2958.2011.07608.x 21401736 PMC3097173

[B65] Wadhams GH Armitage JP Making sense of it all: bacterial chemotaxis Nat Rev Mol Cell Biol 2004 5 12 1024 1037 10.1038/nrm1524 15573139

[B66] Paredes CJ Rigoutsos I Papoutsakis ET Transcriptional organization of the *Clostridium acetobutylicum *genome Nucleic Acids Res 2004 32 6 1973 1981 10.1093/nar/gkh509 15060177 PMC390361

[B67] Ohtani K Hayashi H Shimizu T The *luxS *gene is involved in cell-cell signalling for toxin production in *Clostridium perfringens* Mol Microbiol 2002 44 1 171 179 10.1046/j.1365-2958.2002.02863.x 11967077

[B68] Mitchell Wilfrid J Tangney M Dürre P Carbohydrate uptake by the phosphotransferase system and other mechanisms Handbook on clostridia 2005 London: CRC press 155 175

[B69] Lee J Blaschek HP Glucose uptake in *Clostridium beijerinckii *NCIMB 8052 and the solvent-hyperproducing mutant BA101 Appl Environ Microbiol 2001 67 11 5025 5031 10.1128/AEM.67.11.5025-5031.2001 11679321 PMC93266

[B70] Mitchell WJ Shaw JE Andrews L Properties of the glucose phosphotransferase system of *Clostridium acetobutylicum *NCIB 8052 Appl Environ Microbiol 1991 57 9 2534 2539 1768126 10.1128/aem.57.9.2534-2539.1991 PMC183615

[B71] Barabote RD Saier MH Comparative genomic analyses of the bacterial phosphotransferase system Microbiol Mol Biol Rev 2005 69 4 608 634 10.1128/MMBR.69.4.608-634.2005 16339738 PMC1306802

[B72] Mitchell WJ Tangney M Dürre P Carbohydrate uptake by the phosphotransferase system and other mechanisms Handbook on clostridia 2005 London: CRC press 155 175

[B73] Tatusov RL Galperin MY Natale DA Koonin EV The COG database: a tool for genome-scale analysis of protein functions and evolution Nucleic Acids Res 2000 28 1 33 36 10.1093/nar/28.1.33 10592175 PMC102395

[B74] Reysenbach AL Ravenscroft N Long S Jones DT Woods DR Characterization, biosynthesis, and regulation of granulose in *Clostridium acetobutylicum* Appl Environ Microbiol 1986 52 1 185 190 16347108 10.1128/aem.52.1.185-190.1986 PMC203438

[B75] Yang HH Liu MY Romeo T Coordinate genetic regulation of glycogen catabolism and biosynthesis in *Escherichia coli *via the CsrA gene product J Bacteriol 1996 178 4 1012 1017 8576033 10.1128/jb.178.4.1012-1017.1996 PMC177760

[B76] Stock AM Robinson VL Goudreau PN Two-component signal transduction Annu Rev Biochem 2000 69 1 183 215 10.1146/annurev.biochem.69.1.183 10966457

[B77] West AH Stock AM Histidine kinases and response regulator proteins in two-component signaling systems Trends Biochem Sci 2001 26 6 369 376 10.1016/S0968-0004(01)01852-7 11406410

[B78] Annous BA Blaschek HP Isolation and characterization of *Clostridium acetobutylicum *mutants with enhanced amylolytic activity Appl Environ Microbiol 1991 57 9 2544 2548 1722664 10.1128/aem.57.9.2544-2548.1991 PMC183617

[B79] Qureshi N Lolas A Biaschek HP Soy molasses as fermentation substrate for production of butanol using *Clostridium beijerinckii *BA101 J Ind Microbiol Biotechnol 2001 26 5 290 295 10.1038/sj.jim.7000131 11494105

[B80] Li H Ruan J Durbin R Mapping short DNA sequencing reads and calling variants using mapping quality scores Genome Res 2008 18 11 1851 1858 10.1101/gr.078212.108 18714091 PMC2577856

[B81] Gentleman R Carey V Bates D Bolstad B Dettling M Dudoit S Ellis B Gautier L Ge Y Gentry J Bioconductor: open software development for computational biology and bioinformatics Genome Biol 2004 5 10 R80 10.1186/gb-2004-5-10-r80 15461798 PMC545600

[B82] Anders S Huber W Differential expression analysis for sequence count data Genome Biol 2010 11 10 R106 10.1186/gb-2010-11-10-r106 20979621 PMC3218662

[B83] Robinson MD McCarthy DJ Smyth GK edgeR: a Bioconductor package for differential expression analysis of digital gene expression data Bioinformatics 2010 26 1 139 140 10.1093/bioinformatics/btp616 19910308 PMC2796818

[B84] Eisen MB Spellman PT Brown PO Botstein D Cluster analysis and display of genome-wide expression patterns Proc Natl Acad Sci USA 1998 95 25 14863 14868 10.1073/pnas.95.25.14863 9843981 PMC24541

[B85] Reich M Liefeld T Gould J Lerner J Tamayo P Mesirov JP GenePattern 2.0 Nature Genet 2006 38 5 500 501 10.1038/ng0506-500 16642009

