# Supplementary material for: Genome-wide dynamic transcriptional profiling in clostridium beijerinckii NCIMB 8052 using single-nucleotide resolution RNA-Seq
Source: BMC Genomics. 2012 Mar 20;13:102. doi: 10.1186/1471-2164-13-102 (PMC3395874; doi:10.1186/1471-2164-13-102)
Supplement: Additional file 5 — The qRT-PCR verification of RNA-Seq quantification results for selected genes. The fold change of each gene's expression along time course measured by either qRT- PCR (in black) or RNA-Seq (in red) was compared to that of the same gene at first time point (time point A in Figure 1A). (A) Cbei_4852, pfk. (B) Cbei_1903, fba. (C) Cbei_4851, pyk. (D) Cbei_0203, ptb. (E) Cbei_0204, buk. (F) Cbei_3833, ctfA. (G) Cbei_3834, ctfB. (H) Cbei_0411, thlA. (I) Cbei_0325, hbd. [file 1471-2164-13-102-S5.DOC]

|  |  |  |
| --- | --- | --- |
|  |  |  |
|  |  |  |

**Figure S1**
