# Supplementary material for: Genome-wide dynamic transcriptional profiling in clostridium beijerinckii NCIMB 8052 using single-nucleotide resolution RNA-Seq
Source: BMC Genomics. 2012 Mar 20;13:102. doi: 10.1186/1471-2164-13-102 (PMC3395874; doi:10.1186/1471-2164-13-102)
Supplement: Additional file 6 — RNA-Seq data analysis. [file 1471-2164-13-102-S6.DOC]

## RNA-Seq data analysis.

## Time course RPKM values were first transformed to log_2_-scale. The distribution histogram of the data was shown in Figure S2. The data covered a large range and were normally distributed with center at around 5. For better representation of the data by the heatmap plots, the log_2_-transfromed RPKM values were then centered by subtracting 5. The histogram for the distribution of the “5-centered” data was shown in Figure S3. With a color scale ranging from -5 to 5, the heatmap plots represented most of the transformed gene expression data.

**
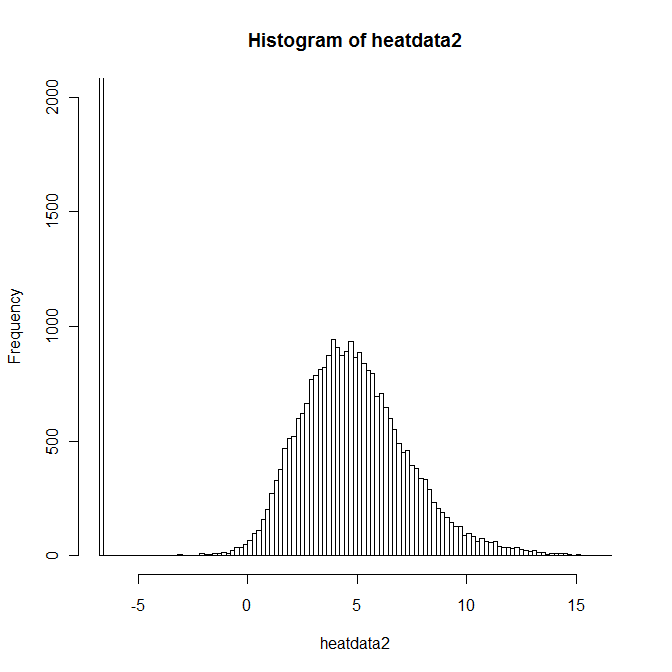
**

**Figure S2 Distribution of the log_2_-tranformed RPKM values for all the genes.**

**
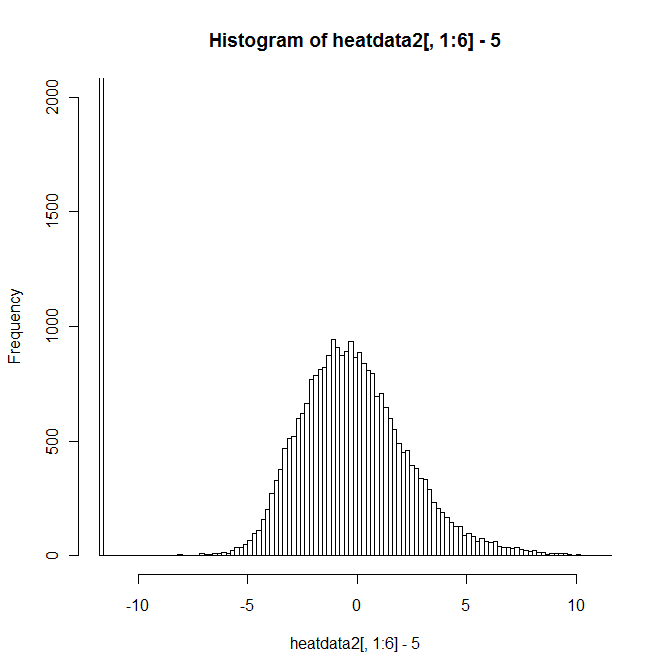
**

**Figure S3 Distribution of the “5-centered” log_2_-tranformed RPKM values for all the genes.**
