# Supplementary material for: Genome-wide dynamic transcriptional profiling in clostridium beijerinckii NCIMB 8052 using single-nucleotide resolution RNA-Seq
Source: BMC Genomics. 2012 Mar 20;13:102. doi: 10.1186/1471-2164-13-102 (PMC3395874; doi:10.1186/1471-2164-13-102)
Supplement: Additional file 7 — Genes and primer sequences for qRT-PCR test. [file 1471-2164-13-102-S7.DOC]

**Table S5** Genes and primer sequences for qRT-PCR test

| No. | Gene ID | Strand | Forward primer (5'-3') | Reverse primer (5'-3') | Size of amplicon (bp) | Gene product description |
| --- | --- | --- | --- | --- | --- | --- |
| 1 | Cbei_0203 | + | T G A T G G A C C T T T T G C C T T A G A T | T T T C G A A T G A A T C T G C T C T T G A | 227 | phosphate butyryltransferase |
| 2 | Cbei_0204 | + | A T A T G G C A A G G C T G T A G G A A A A | A A G G T C T G G A A C A A G T G T T G G T | 235 | butyrate kinase |
| 3 | Cbei_0325 | + | T C C A A T G G G A C C T T T A G C T T T A | T C C T T T T C C T G A T T T T C T T C C A | 163 | 3-hydroxybutyryl-CoA dehydrogenase |
| 4 | Cbei_0411 | + | T G G A C C A T T C C A T G C A A C T A | T G C T A A G C T T T G A G C A G C A A | 106 | acetyl-CoA acetyltransferase |
| 5 | Cbei_1903 | - | A G C A G A A G T T G G T T C A A T T G G T | T G A A T G T T T C C A A T A C C A G C A G | 132 | fructose-1,6-bisphosphate aldolase, class II |
| 6 | Cbei_3833 | + | T C C C C A C A A G G A A C A C T G A | T T T G C A G C C A T T G C C A T A | 257 | 3-oxoacid CoA-transferase, A subunit |
| 7 | Cbei_3834 | + | T G T T G C T G T T C T T G G T G C T C | T T G C G C C T A T T G C T A A A T C C | 116 | 3-oxoacid CoA-transferase, B subunit |
| 8 | Cbei_4851 | - | A T G G A G A C C A T G A A G A A C A C G | T C C T G G C T T A A C A T C A T T T G C | 251 | pyruvate kinase |
| 9 | Cbei_4852 | - | C A A G C T T A T G G T G T T G A A G C T C T | A T A A A T C T C C G C A G T C T C T A C C C | 262 | 6-phosphofructokinase |
| 10 | Cbei_2428* | + | A C A G A T G G A A C A T C A T T G C T T G | A T C A A A T A A A T G C G C T C C A A G T | 159 | peptidase T |

*Cbei_2428 was selected as the endogenous control gene.
